# The Coffee Heart

**Published:** 1902-02

**Authors:** 


### The Coffee Heart.

According to the *Dietetic and Hygienic Gazette,* the life insurance societies in the United States are beginning to realize that excessive coffee drinking by Americans is increasing the risks of life. Therefore, the term "coffee heart" has already been added by some of them to their regular classification of the functional derangements of the heart. The effect of coffee upon the heart is more lasting and therefore worse than the effects of excessive liquor drinking. R.

